# The Role of Chaperone-Mediated Autophagy in Cell Cycle Control and Its Implications in Cancer

**DOI:** 10.3390/cells9092140

**Published:** 2020-09-22

**Authors:** Marina Andrade-Tomaz, Izadora de Souza, Clarissa Ribeiro Reily Rocha, Luciana Rodrigues Gomes

**Affiliations:** 1 Departamento de Oncologia Clínica e Experimental, Escola Paulista de Medicina, Universidade Federal de São Paulo, São Paulo 04037-003, SP, Brazil; marina.tomaz@unifesp.br (M.A.-T.); izadora.souza@unifesp.br (I.d.S.); clarissa.rocha@unifesp.br (C.R.R.R.); 2 Laboratório de Ciclo Celular, Center of Toxins, Immune Response and Cell Signaling (CeTICS), Instituto Butantan, São Paulo 05503-001, SP, Brazil

**Keywords:** autophagy, chaperone-mediated autophagy (CMA), cell cycle, cancer, checkpoints, MYC, hypoxia-inducible factor-1 subunit alpha (HIF-1α), checkpoint kinase 1 (CHK1)

## Abstract

The cell cycle involves a network of proteins that modulate the sequence and timing of proliferation events. Unregulated proliferation is the most fundamental hallmark of cancer; thus, changes in cell cycle control are at the heart of malignant transformation processes. Several cellular processes can interfere with the cell cycle, including autophagy, the catabolic pathway involved in degradation of intracellular constituents in lysosomes. According to the mechanism used to deliver cargo to the lysosome, autophagy can be classified as macroautophagy (MA), microautophagy (MI), or chaperone-mediated autophagy (CMA). Distinct from other autophagy types, CMA substrates are selectively recognized by a cytosolic chaperone, one-by-one, and then addressed for degradation in lysosomes. The function of MA in cell cycle control, and its influence in cancer progression, are already well-established. However, regulation of the cell cycle by CMA, in the context of tumorigenesis, has not been fully addressed. This review aims to present and debate the molecular mechanisms by which CMA can interfere in the cell cycle, in the context of cancer. Thus, cell cycle modulators, such as MYC, hypoxia-inducible factor-1 subunit alpha (HIF-1α), and checkpoint kinase 1 (CHK1), regulated by CMA activity will be discussed. Finally, the review will focus on how CMA dysfunction may impact the cell cycle, and as consequence promote tumorigenesis.

## 1. Introduction

Cancer cells usually present a variety of impaired cellular mechanisms as a consequence of genomic instability. However, changes in cell cycle machinery functionality are the primary reason why cancer emerges, grows, and spreads. Most of the cancer hallmarks are sustained by the ability of cancer cells to constantly proliferate. Therefore, the starting point of understanding carcinogenesis is the comprehension of how and why the cell cycle is reprogrammed to prioritize proliferation, even under environmental stress and DNA damage conditions. In a tumorigenic context, cells acquire their own mechanisms to reach enough mitogenic stimulation for exit quiescence. Furthermore, the cell cycle checkpoints, which ensure proper environmental conditions and DNA integrity during the cellular division process, are commonly disrupted [1,2]. As a central core process in cancer development, cell cycle modulation by chemotherapy is a well-established clinical strategy. Nevertheless, due to increased drug resistance reported in clinical oncology, new targets that could affect cell cycle progression, either by direct or indirect mechanism, are currently being investigated [3,4].

Each step of the cell cycle is carefully coordinated by the dynamics of specific cyclins and cyclin-dependent kinase (CDK) complexes, which are regulated by tumor suppressor genes, such as p53, p73, and RB [5,6,7]. One of the possible mechanisms to activate cyclin production and trigger cell proliferation cascades is by the phosphorylation of β-catenin, allowing pyruvate kinase isozyme M2 (PKM2) binding. The β-catenin–PKM2 complex interacts with transcription factor 4 (TCF4), promoting the transcription of MYC, a proto-oncogene, and cyclin D1, which binds to cyclin-dependent kinase 4/6 (CDK 4/6) and leads the cell to gap 1 (G1) phase, the first stage of the cell cycle [8]. During G1, cyclin–CDK gradually phosphorylates RB until its dissociation from E2F, allowing the cell to progress to the synthesis phase (S phase), the next stage of the cell cycle [9]. After genetic content is replicated and checked for errors, the cell enters gap 2 (G2) phase, divides its organelles and prepares to complete cell division at mitosis (M) phase. 

To ensure the cell does not replicate under unfavorable environmental conditions or carrying DNA damage, the cell cycle may be arrested, in three specific checkpoints: G1 to S, G2 to M, and at the M phase. Checkpoint kinase 2 (CHK2) activation can induce checkpoints at the G1 to S and G2 to M transitions by phosphorylating p53, which leads to cell cycle arrest through the activation of CDK inhibitor p21. Similarly, checkpoint kinase 1 (CHK1) also participates in the regulation of the cell cycle, either through p53 or protein cell division cycle 25 (CDC25) phosphorylation. When the primary cause of cell cycle arrest persists, p53 can drive the cell into the apoptotic cell death pathway, in order to prevent malignant transformation [5,9]. Nevertheless, cancer cells present mechanisms to reprogram the regulation of the cell cycle checkpoints, and therefore enables these cells to proliferate carrying DNA lesions and despite poor nutrient and oxygen availability.

Modulation of the levels of cell cycle regulators is a common tool used by cancer cells to reprogram cellular division. Thus, a decrease of tumor-suppressors, such as p53 and RB proteins, or an increase of proto-oncogene levels, for example MYC and epidermal growth factor receptor (EGFR), are frequent events in primary tumors [10,11]. Therefore, the balance between synthesis and degradation of these cell-cycle proteins can determine proliferation rates. Protein degradation is performed by the ubiquitin–proteasome system (UPS) and autophagy. However, autophagy goes far beyond protein degradation, and it is not just a mechanism for controlling the quantity of these biomolecules. Autophagy is the main catabolic system in eukaryotic cells, capable of the degradation of biomolecules (proteins, lipids, carbohydrates, and nucleic acids), organelles, and pathogens, through both selective and unselective mechanisms [12,13]. As an essential process for the maintenance of cellular homeostasis and health, autophagy also contributes to genomic integrity and tumor suppression.

**Figure 1:**
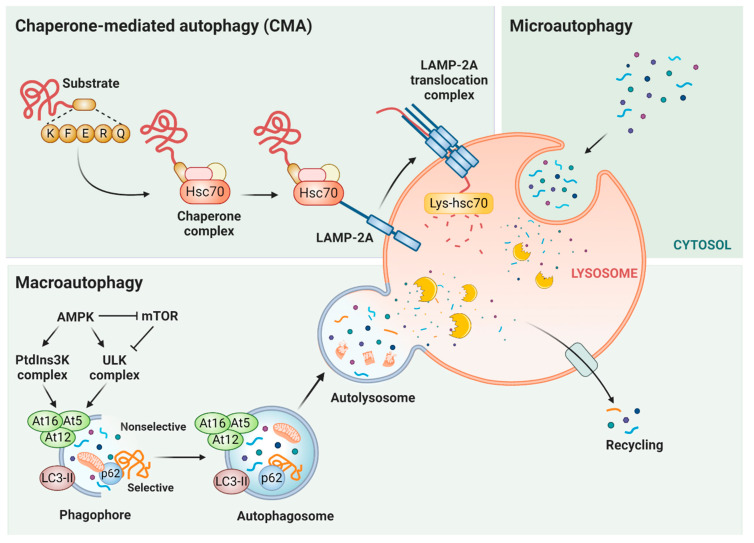
Main types of autophagy. There are three forms of autophagy: chaperone-mediated autophagy (CMA), microautophagy, and macroautophagy. CMA is a selective type of autophagy, in which target proteins with a KFERQ sequence are recognized by cytosolic chaperone HSC70 and co-chaperones (chaperone complex). Subsequently, these CMA substrates are taken one-by-one to lysosome-associated membrane protein type 2A (LAMP-2A) and translocated inside the lysosome through the action of the LAMP-2A translocation complex and the chaperone located in the lysosome lumen (Lys-HSC70). In microautophagy, the substrate capture occurs directly by lysosomal membrane invagination and rearrangement. Macroautophagy can be both a selective and nonselective kind of autophagy. MA is controlled by the mammalian target of rapamycin (mTOR) and AMP-activated protein kinase (AMPK), which inhibits mTOR and stimulates unc-51-like autophagy-activating kinase (ULK) and class III phosphatidylinositol 3-kinase (PtdIns3K) complexes. Further, the phagophore formation is concluded by light chain 3 (LC3) and autophagy-related gene 12 (Atg12) systems. Then the authophagosome can fuse with the lysosome (to form an autolysosome) for substrate degradation and later recycling of metabolic precursors. In nonselective macroautophagy, the substrates are carried to degradation in the lysosome through autophagosomes. In selective macroautophagy, there are receptors like p62 that recognize the cargo proteins and target them to the autophagosome. Figure created with Biorender.

There are three distinct autophagy pathways: macroautophagy (MA), microautophagy (MI), and chaperone-mediated autophagy (CMA) (Figure 1). The canonical process of MA is triggered by the inactivation of the mechanistic target of the protein complex mammalian target of rapamycin complex 1 (mTORC1), which culminates in the activation of multiprotein complexes for phagophore formation. Proteins belonging to the autophagy-related gene (ATG) family promote the maturation of phagophore into autophagosome, a double-membrane vesicle capable of fusing with lysosomes. This fused organelle, called an autolysosome, is an acidic vesicle that allows lysosomal hydrolases to degrade the autophagy substrates [14,15]. Regarding the MA function in tumor progression, this type of autophagy displays a context-dependent role. Thus, under physiological circumstances, as a major mechanism of cytoprotection, MA provides better outcomes for normal cells under stress conditions, preventing genomic instability. However, once malignancy is settled, MA turns into an ally for cancer cell metabolism. Therefore, MA can protect tumors against cytotoxic agents, being able to assist in the maintenance of cancer stem cells, tumor proliferation, metastatic recurrence, and the development of resistance to antineoplastic agents [16,17,18,19,20,21,22]. The participation of MA in cell cycle regulation occurs through selective degradation, mediated mostly by p62, which targets proteins involved in cellular proliferation, checkpoint regulation, DNA damage responses, and senescence [23,24,25,26]. 

In MI, the cytosolic cargo is captured directly through the formation of small vesicles, by invagination of the lysosomal membrane. However, the regulation of MI is still unclear in eukaryotic cells, and nothing is known about its potential role in cancer. Vesicle integration is not the only manner by which cargo can be delivered to the lysosome for degradation. The substrate (proteins specifically) to be degraded can also be selectively recognized and carried to the lysosome through CMA. In this selective type of autophagy, which is only present in mammals and birds, the substrate is identified by a cytosolic chaperone and delivered to the lysosomal surface, and upon its unfolding, can be internalized through a membrane translocation complex, all without the participation of vesicles.

This autophagy mechanism, coined CMA 20 years ago by Cuervo and Dice, has been shown to play fundamental roles in aging and metabolism [27,28,29]. Since the publication of the results by Kon et al., a series of evidence has accumulated showing the important role played by CMA in the biology of cancer, both in early and in more aggressive stages [30]. Moreover, due to its selective and timely degradation mechanism, CMA also proved to be an important pathway that controls cell cycle regulator levels [31,32,33,34,35]. Thus, in this present work, we review the potential influence of CMA in the cell cycle in the cancer context and the mechanisms through which this modulation occurs, as well as discuss the significant gaps in the comprehension of CMA–cell cycle interaction open to be further explored.

## 2. Chaperone-Mediated Autophagy (CMA)

CMA is an intracellular catabolic pathway that mediates the degradation of soluble cytosolic proteins in lysosomes [36]. In contrast to macroautophagy and microautophagy, where the substrate is delivered to lysosomes inside vesicles, CMA protein targets are recognized one-by-one by the cytosolic chaperone Hsc70, which along with its modulatory co-chaperones brings them to the lysosome’s surface [36]. CMA selectivity is conferred by a specific sequence (KFERQ-like motifs) present in all CMA target proteins. The CMA motif is based on the charge of the amino acids, so in certain cases, it is possible to obtain a recognizable motif—even if it is incomplete—through post-translational modifications, such as phosphorylation or acetylation [37].

After this targeting step, the substrate interacts with the cytosolic tail of the lysosome-associated membrane protein type 2A (LAMP-2A), which acts as a receptor for the CMA pathway [36,38]. This protein is a spliced variant of the *Lamp2* gene. The other two variants (LAMP-2B and LAMP-2C) have different transmembrane and cytosolic tail regions, but share a common luminal domain [36,37]. The substrate binding to the LAMP-2A monomer triggers the formation of a 700 kDa, multimeric complex at the lysosomal membrane to mediate its translation. Chaperones participate in several steps of this pathway, hence the motivation for the name CMA [27]. Besides the cytosolic chaperone Hsc70, which plays a crucial role in recognizing CMA cargo and delivery to lysosome, there is also a lysosomal form of Hsc70 (lys-Hsc70) that is essential for the translocation of the substrate protein across the lysosomal membrane. Moreover, Hsp90, present in the luminal part of the lysosome membrane, stabilizes the conformational changes that LAMP-2A undergoes during its transition from the monomer to the multimer stage [39]. The presence of Hsp90 in the cytosol, close to the lysosomal surface, is also required, since this chaperone binds to substrate proteins during the unfolding step that precedes translocation, in order to avoid undesirable interactions [40,41]. After translocation, the substrate reaches the lysosomal matrix, where it undergoes a complete degradation, and LAMP-2A is rapidly disassembled from the translocation complex into monomers, allowing the binding of new substrates [39].

## 3. Physiological and Pathological Roles of CMA

Quality control of cellular components is an important function of CMA, since it is able to selectively remove damaged or misfolded proteins. Consequently, CMA performs a key role in response to several stressors that generate protein damage, particularly oxidative stress. CMA is upregulated in response to oxidative stress, and a failure in its upregulation leads to accumulation of oxidative damage and results in reduced cellular viability [42,43]. CMA is also induced in other conditions, such as exposure to denaturing toxic compounds and hypoxia [44,45].

Another central role of CMA is in the control of cellular energy homeostasis. During prolonged starvation, CMA is maximally activated, degrading proteins that are no longer needed, and thus providing free amino acids used in the synthesis of essential proteins. Thus, nutrient deprivation is the classical approach for CMA activation [46]. Therefore, CMA allows cellular growth and survival under low-nutrient conditions. On the other hand, CMA is inhibited by chronic exposure to a high-fat diet, probably due to the decrease in LAMP-2A proteins in the lysosomes [47]. 

It has been known for a long time that some glycolytic enzymes are CMA substrates [48]. However, the physiological relevance of CMA and its impact on metabolism in vivo has only recently been revealed [28]. By the generation of conditional knockout mouse to selectively block CMA in liver, it was found that the loss of CMA leads to profound changes in hepatic carbohydrate and lipid metabolism. These alterations have an impact on the energetic balance of the whole organism [28]. Comparative proteomics revealed that key enzymes in carbohydrate and lipid metabolism are degraded by CMA [28]. Also related to lipid metabolism, CMA has been recently demonstrated as essential for lipolysis [49]. Although CMA is not able to degrade lipids, the blockage of CMA in the liver leads to steatosis [28]. Intracellular lipids are stored in lipid droplets (LDs), which are enclosed by structural proteins of the perilipin (PLIN) family: PLIN1, PLIN2, and PLIN3 [50]. It has been demonstrated that CMA degrades PLIN2 and PLINConsequently, CMA blockage results in impaired lipolysis [49].

Given its selectivity, CMA exerts numerous functions, due to the proteins it is able to degrade, and consequently, which molecular pathways it is able to interfere. Through the degradation of IκB, CMA controls transcription in response to nutrient stress mediated by NF-κB [51]. The transcription factor PAX2 is also a CMA target, and its degradation allows the regulation of cell growth [52]. CMA is also involved in regulation of the adaptive immune system [20]. Itch and RCAN1, negative regulators of T cell receptor (TCR) signaling and necessary for full T cell activation, are degraded by CMA. Consequently, activation of CMA facilitates activation-induced T cell responses [53]. 

Increasing evidence has shown that a malfunction of CMA plays a key role in several human disorders [28,30,37,41]. Both the increase and decrease in CMA activity have been associated with diseases. In general, in neurodegenerative pathologies, there is a failure of the proteolytic systems, leading to the accumulation of deleterious proteins [54]. In this sense, CMA is impaired in both familial and sporadic Parkinson’s disease (PD) [55,56]. Leucine-rich repeat kinase 2 (LRRK2) and α-synuclein, the two most commonly mutated proteins in patients with familial PD, are degraded by CMA [55,56,57,58]. However, mutant variants of LRRK2 and α-synuclein, despite being recognized by Hsc70 and delivered to the lysosome, fail to reach the lysosomal lumen. Due to abnormal interactions of these toxic mutant proteins with LAMP-2A, the internalization of mutant LRRK2 and α-synuclein is obstructed. The aberrant high affinity of these mutant proteins with the CMA translocation complex not only inhibits their own degradation, but also prevents the degradation of other CMA substrates [37,55,56]. 

CMA is also associated with Alzheimer’s disease and tauopathies [59]. Wild-type tau undergoes degradation by CMA, while mutant tau displays distinct processing by CMA. After binding to LAMP-2A, the mutant tau protein is only partially internalized and cleaved, resulting in the formation of amyloidogenic tau fragments at the lysosomal membrane [37,59]. Oligomerization of these fragments causes a rupture of the lysosomal membrane, which, consequently, leads to CMA blockage and allows tau aggregation, now in the cytosol; in addition, the aggregates can act as a nucleating centers [37,59]. Furthermore, CMA is also able to degrade the regulator of calcineurin 1 (RCAN1), a protein linked to neuronal death and frequently highly expressed in the brains of patients with Alzheimer’s disease [60]. 

A decline in CMA also occurs in physiological aging, and it is probably caused by changes in the lysosomal membrane lipid constituents. This alters LAMP-2A stability, which, consequently, leads to the enhancement of LAMP-2A degradation in the lysosomal lumen, reducing CMA activity in older organisms [47,61]. Given the CMA key role in protein quality control, the direct consequence of CMA failure is deficiency with regard to the removal of damaged proteins and the capacity to respond to stressors [37].

## 4. CMA’s Role in Cancer

Initially, CMA has been linked to pro-tumorigenic functions, since upregulation of this autophagy pathway is associated with positive modulation of tumor cell survival and growth [30]. Subsequent studies have demonstrated that CMA plays a more complex and context-dependent role. In fact, with rare exceptions, it has been confirmed that cancer cells from different tissues and tumor stages have upregulated CMA activity [62,63,64]. However, in non-tumorigenic cells, CMA has an antitumor function, preventing malignant transformation [31].

### 4.1. Anti-Tumor Functions of CMA

Physiological anti-tumor functions of CMA are described above. Studies on aging using mice defective in CMA only in the liver display a higher incidence of spontaneous hepatic tumors with age [29]. Due to its selectivity, CMA is able to control the levels of specific proteins, including proto-oncogenic proteins, such as mouse double-minute 2 homolog (MDM2) and the translationally-controlled tumor-associated protein TCTP [65,66]. Therefore, a malfunction of CMA would lead to the accumulation of these oncogenic proteins. Although MYC is not a CMA target, MYC degradation levels are also controlled by CMA, but through an indirect mechanism [31]. In fact, CMA degrades cancerous inhibitors of protein phosphatase 2A (CIP2A), a protein that stops MYC degradation by the proteasome. Higher levels of MYC protein in CMA-deficient cells lead to higher proliferation capacity, pronounced capability of soft-agar colony formation, and tumor-favorable metabolic changes. Therefore, by preventing MYC accumulation, CMA has proven able to preclude malignant transformation [31]. 

### 4.2. Pro-Tumor Functions of CMA

The anti-tumor function of CMA becomes pro-tumor in cancer cells [41]. After malignant transformation, CMA becomes highly active to sustain important pro-oncogenic functions [41]. CMA is upregulated in different cancer cell lines, and inhibition of CMA in these cells has resulted in both increased cell death and decreased proliferation rates [30]. Moreover, cancer cells deficient for CMA showed decreased glycolytic capability, which correlates with a significant reduction of mRNA levels of some glycolytic enzymes [30]. CMA blockage in cancer cells has also limited their proliferative capacity in vivo, reduced the number of metastases and induced regression of existing human lung cancer xenografts in mice [30]. 

Changes in cellular metabolism through a switch from oxidative phosphorylation to aerobic glycolysis (known as the Warburg effect) are typical of the tumor transformation process [67]. Glycolytic enzymes are bona fide CMA substrates, which indicates CMA’s role in glycolysis control. The acetylated form of the embryonic isoform M2 of pyruvate kinase (PKM2) is degraded by CMA. Ectopic expression of an acetylation mimetic mutant of PKM2, not degradable by CMA, accumulates glycolytic intermediates and promotes cell proliferation and tumor growth [68]. Hexokinase II, a key glycolytic enzyme required for tumorigenesis, is also a CMA target [69]. However, when phosphorylated at Thr473, hexokinase II is not degraded by CMA, increasing its stability. The increased hexokinase II enzymatic activity enhances glycolysis and the growth of breast cancer cells both in vitro and in vivo [70]. 

CMA also has an impact in tumor immunology. The interaction of tumor cells with pericytes (PCs), perivascular stromal cells, contributes to immunotolerance, allowing tumor growth. Using a glioblastoma cell model, it was demonstrated that these cancer cells induce the upregulation of CMA in PCs. This is necessary for maintaining an anti-inflammatory phenotype that precludes T cell activation for tumor clearance [41,71]. CMA inhibition in PC promotes the death of glioblastoma cells, and in vivo, CMA-defective PCs have shown decreased glioblastoma proliferation and effective immune response [71]. 

## 5. CMA Control of Cell Cycle in Distinct Cellular Contexts

**Figure 2:**
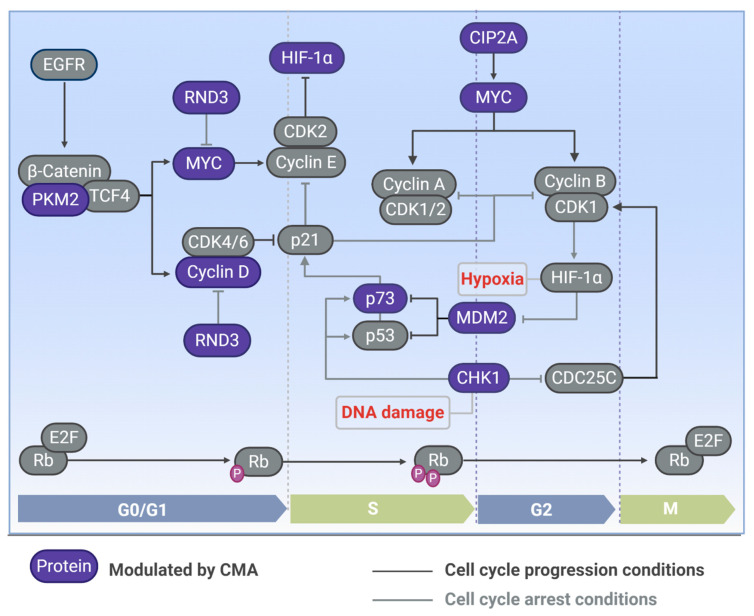
Cell cycle proteins modulated by CMA. CMA modulates the levels of proteins involved in the cell cycle, such as pyruvate kinase (PKM2), which stimulates MYC and cyclin D for cell cycle progression in G1. Cyclin D interacts with cyclin-dependent kinase (CDK) 4/6 and inhibits p21 for cell cycle progression; p21 can also be modulated by Rho family GTPase 3 (RND3), another CMA substrate that induces cell cycle arrest in G1. MYC is also induced through the cancerous inhibitor of protein phosphatase 2A (CIP2A), which is modulated by CMA, and is inhibited by RDN3. MYC is required for positive cell cycle regulation and progression through the cyclin-CDK induction. Checkpoint kinase 1 (Chk1) induces cell cycle arrest in DNA damage conditions, and it is degraded by CMA. Mouse double-minute 2 homolog (MDM2) is a CMA substrate that inhibits p53 and p73 for cell cycle progression; p73 is also controlled by CMA and promotes cell cycle arrest by inducing p21. In hypoxia situations, the hypoxia-inducible factor-1 subunit alpha (HIF-1α) is induced by CDK1 and inhibits MDM2 to trigger cell cycle arrest, HIF-1α can be inhibited by CDK2 for cell cycle progression. Proteins reported to be modulated by CMA are represented in blue. Phases of the cell cycle: G1 (gap 1 phase), S (synthesis phase), G2 (gap 2 phase), M (mitosis phase). Figure created with Biorender.

Potentially more than one third of the cytosolic proteins carry a KFERQ-like motif [72]. Thus, by controlling protein degradation, and consequently, the number of specific proteins, CMA is able to regulate several cellular mechanisms. Kirchner and colleagues performed a proteome-wide study on KFERQ-like motifs in the human proteome [72]. Besides determining data about abundance, location, composition, and evolutionary conservation, they analyzed whether KFERQ-like motifs associate with particular cellular processes by performing an enrichment analysis, using biological annotations from gene ontology [72]. Thus, it was demonstrated that several cellular functions, including the cell cycle, formed clusters associated with all kinds of KFERQ-like motifs (canonical and phosphorylation- or acetylation-generated motif). The role of CMA in cell cycle control is still little explored. However, the involvement of CMA in the control of some important cell cycle modulators has already been demonstrated (as indicated in Figure 2).

### 5.1. Tumor Transformation: MYC

CMA controls MYC levels through an indirect mechanism [31]. MYC is a transcription factor of the helix–loop–helix leucine zipper family, which is involved in the regulation of many target genes, both by activation and repression [73]. It is able to stimulate cell cycle progression through many mechanisms, including positive regulation of the most critical positive cell cycle modulators [74]. Thus, key cell cycle regulators are encoded by MYC target genes, such as cyclins (cyclin A, cyclin B1, D-type cyclins, and E-type cyclins), CDKs (CDK1, 2, 4, 6) and E2F transcription factors (E2F1, 2, 3) [73]. Besides its direct effect on transcription, MYC also positively regulates the cell cycle by controlling the activities of the cyclin/CDK complex thorough the induction of CDK-activating kinase (CAK) and CDC25 phosphatases. Moreover, MYC also stimulates the cell cycle by antagonizing the activity of cell cycle inhibitors, such as p15, ARF, p21, and p27 through distinct mechanisms [74]. Since the hyperstimulation of the cell cycle is vital in the process of neoplasia development, MYC overexpression is commonly detected in human tumors. Thus, about 60–70% of human solid and hematopoietic tumors present overexpressed MYC [73]. 

To better understand the role of CMA in the first steps of tumor development, a recent study addressed whether CMA interferes in oncogene-driven malignant transformation [31]. Thus, it was demonstrated that CMA inhibition in fibroblasts enhances the efficiency of cellular transformation mediated by MYC. Besides the augmentation of cell proliferation and colony formation in semi-solid substrates, CMA blockage accentuated tumorigenesis-related metabolic changes commonly related to tumor transformation. Therefore, the blockage of CMA potentiated MYC-driven changes in oxygen consumption and extracellular acidification, contributing to the switch from oxidative phosphorylation to aerobic glycolysis [31]. 

Given the evident increase in MYC levels in CMA-deficient cells, it was investigated whether MYC is degraded by CMA. The results suggest that neither endogenous nor exogenously expressed MYC proteins are targeted for CMA degradation in fibroblasts [31]. In fact, it was already known that MYC is mostly degraded by the ubiquitin–proteasome system (UPS), and that its recognition by the UPS machinery is controlled by phosphorylation of specific sites [75]. When phosphorylated at Ser62 MYC is stable, and therefore is not degraded by UPS. The first step for MYC degradation consists of its phosphorylation at Thr58, mediated by glycogen synthase kinase 3 beta (GSK3β). The definitive step for MYC UPS targeting involves its dephosphorylation at the Ser62 site, mediated by the protein phosphatase 2A (PP2A). Therefore, only after removal of Ser62 is MYC recognized by the E3 ligase, which ubiquitinates MYC and targets it for proteasomal degradation [75]. MYC proteasome-dependent degradation is compromised under CMA-deficient conditions [31]. Furthermore, MYC phosphorylation at Ser62 proved to be essential for MYC protein accumulation mediated by CMA blockage, since expression of mutated MYC at this specific phosphorylation site did not present augmented levels of MYC. 

Higher levels of Ser62-phosphorylated MYC in CMA-deficient cells has been shown to be correlated with reduced PP2-mediated dephosphorylation [31]. CIP2A, the guardian of MYC, which stabilizes this oncoprotein by its inhibitory effect on PP2A, also accumulates in CMA-deficient cells. It was demonstrated that CIP2A is a CMA substrate, and therefore, a fraction of CIP2A undergoes lysosomal degradation by CMA. Thus, CMA determines MYC protein levels by controlling MYC Ser62 phosphorylation levels and its subsequent delivery to the proteasome [31]. By controlling MYC stability, CMA potentially exerts a key role in the regulation of the cell cycle and cell proliferation, and consequently, in tumor development. 

### 5.2. Hypoxia: HIF-1α

Data published by Hubbi and colleagues suggest that hypoxia-inducible factor-1 subunit alpha (HIF-1α) is a CMA substrate [32]. It is one of the subunits that make up the HIF-1 heterodimer, a transcription factor crucial for mediating the adaptive response to hypoxia. Cell cycle arrest and proliferation inhibition are key adaptive responses to oxygen deprivation [76]. The inhibition of H1F-1α results in the ablation of hypoxia-induced G1 arrest [77]. The HIF-1α-dependent cell cycle arrest is correlated with an increase in the expression of the CDK inhibitors p21 and p27, and also with the hypophosphorylation of RB in a p53-independent manner [77]. Thus, the overexpression of HIF-1α is enough to arrest the cell cycle in G1 phase [76,78].

Pharmacological inhibitors of lysosomal degradation increased both HIF-1 protein levels and activity, whereas macroautophagy blockage did not increase HIF-1 activity [32]. In turn, pharmacological stimulation of CMA led to a reduction of these HIF-1α parameters. Furthermore, it has been demonstrated that HIF-1α interacts with core components of the CMA machinery. Overexpression of either HSC70 or LAMP-2A decreased HIF-1α, whereas knockdown of these CMA-related molecules had the opposite effects. Moreover, CMA and lysosomal biogenesis are induced by hypoxia as part of a negative feedback loop, as observed in cancer cells [32]. Finally, the lysosomal degradation of HIF-1α, mediated by CMA, is regulated by CDK1 and CDK2 through their physical interaction with HIF-1α [76]. 

### 5.3. DNA Damage Response: CHK1

Checkpoint kinase 1 (CHK1) is another CMA substrate essentially involved in cell cycle regulation [33]. The phosphorylation of specific sites is associated with CHK1 activation. Thus, active CHK1 is able to regulate both normal and DNA damage-induced cell cycle arrest [79]. In DNA damage responses (DDRs), CHK1 works by blocking the G2/M transition through the phosphorylation of key regulators of CDK1, which results in CDK1 inactivation. Thus, CHK1 induces DDR cell cycle arrest through the phosphorylation of cell division cycle 25 (CDC25), WEE1 kinase, and polo-like kinase 1 (PLK1). There are three isoforms (A, B, and C) of CDC25 in mammalian cells, which after being phosphorylated by CHK1, act in distinct mechanisms to arrest the cell cycle, all of which culminate in the regulation of CDK1 [79]. Both CDC25 phosphatases and WEE1 kinases interact with CDK1, activating and inhibiting it, respectively. PLK1 can also activate CDK1 through an indirect mechanism, by inhibiting WEE1, besides being able to directly promote cell cycle progression [79].

CHK1 also plays central roles in the unperturbed cell cycle, mediating checkpoints in S and M phases, besides regulating the G2/M transition [79]. In the S phase, CHK1 stimulates cell cycle arrest by inducing CDC25A degradation, which in turn results in CDK2 inhibition. Activation of the cyclin B–CDK1 complex is required for mitotic entry. In the late G2 phase, the formation of this complex can be prevented by CHK1, through the phosphorylation and inactivation of CDC25B phosphatase, and the consequent suppression of CDK1 [79]. 

From the relationship between CMA and CHK1, a novel role for CMA in the maintenance of cellular genome stability has been proposed [33]. CMA is upregulated in response to DNA damage, and its blockage increases cellular susceptibility to genotoxic stress. Thus, failure of CMA activation leads to DNA damage accumulation. CHK1, the serine/threonine protein kinase crucial for mediating DDR, as a CMA substrate, accumulates in cells with defective CMA. CMA blockage modifies the dynamics of CHK1. Therefore, a consequence of CMA inhibition is a sustained CHK1 presence in the nucleus, which in turns leads to DNA damage accumulation and alterations in the levels and phosphorylation status of nuclear proteins of the DNA repair machinery. Among these proteins, the MRE11–RAD50–NBS1 (MRN) complex stands out, as it is involved in the initial processing of double-strand breaks [33].

### 5.4. Different Tumor Cell Models: p73, RND3, and Cyclin D1

Some important molecules involved in cell cycle regulation are controlled by the activity of CMA in tumor cells. Of these, p73, belonging to the p53 family of tumor suppressors, is an example of such regulation [34]. This transcription factor is involved in mediating cellular response to a variety of stressors by inducing several protective mechanisms, among which cell cycle arrest stands out. A fraction of p73 is degraded by UPS, but MDM2 is not the E3 ubiquitin ligase responsible for p73 proteasomal targeting. In fact, MDM2 can interact with p73, not to promote its ubiquitination, but to suppress p73’s transcriptional activity. Recent work has indicated that nerve growth factor receptor (NGFR), a transmembrane receptor intricate in nervous system development, is involved in p73 degradation mediated by CMA [34]. NGFR is highly expressed in several types of cancer, probably as a consequence of its negative effect on p53 activity, since it is able to enhance p53 ubiquitination by MDM2. Thus, Nguyen et al. describe the mechanism of a negative feedback loop in which p73 induces NGFR transcription, and thus stimulates p73 degradation via CMA [34].

Rho family GTPase 3 (RND3), a member of the RND subfamily of the Rho GTPases, is also degraded by CMA in gastric cancer cell lines [35]. Several reports have demonstrated that RND3 is able to inhibit proliferation through cell cycle control. Thus, while RND3 downregulation induces cell cycle progression, its upregulation promotes cell cycle arrest at the G0/G1 phase. RND3 overexpression mediates the increase of p27 and also the decrease of cyclin D1 molecules that are central for the cell cycle machinery. Furthermore, RND3 decreases MYC expression and reduces its transcriptional activity [80]. Villalonga and colleagues demonstrated that RND3 inhibits eIF4E function by blocking its release from 4E–BP1, which consequently contributes to RND3-mediated cell cycle arrest [80]. In glioblastoma cells, RND3 is also involved in the inhibition of cell proliferation by reducing ERK activation, cyclin D1 expression, and RB inactivation [81]. RND3 stimulation obstructs serum-induced cell cycle S phase entry [82]. Moreover, the expression of human papillomavirus E7, adenovirus E1A, and cyclin E rescue cell cycle progression in cells expressing RND3, which therefore suggests that RND3 inhibits the cell cycle upstream of the RB checkpoint [35,82].

Finally, there is an inverse correlation between cyclin D1 expression levels and CMA activity [83]. There is still a long way to go to actually prove that cyclin D1 is a CMA substrate, but whether this modulation occurs through a direct or indirect mechanism, the fact is that CMA affects cyclin D1 levels. Through the inhibition of cyclin D1, a protein essentially involved in cell cycle progression by binding to CDK4/6, CMA is potentially intricate in G0/G1 phase progress control in hepatocellular carcinoma cells [83]. 

## 6. Conclusions and Perspectives

**Figure 3:**
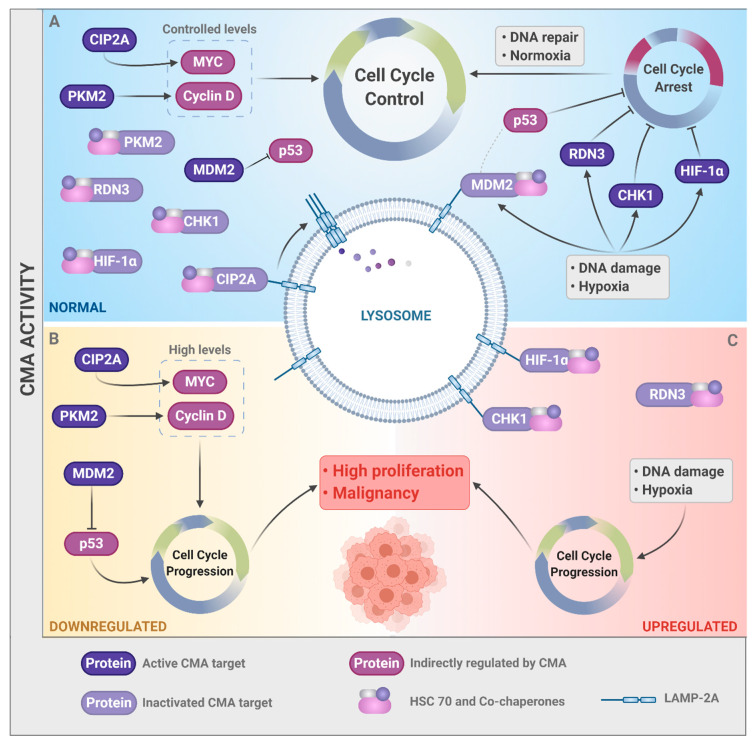
CMA activity controls the cell cycle and cancer. (**A)** In physiological conditions, CMA controls the levels of both positive (e.g., MYC and Cyclin D1) and negative (e.g., RDN3, CHK1, and HIF-1α) cell cycle regulators. (**B)** With low CMA activity, CIP2A and MDM2 are not degraded, which consequently increases the levels of MYC and decreases those of p53, promoting high proliferation and an increase in genetic instability. (**C**) High CMA activity leads to excessive degradation of the negative regulators of the cell cycle under stressful conditions (CHK1, HIF-1α, and RND3), leading to disorderly growth. (**B**,**C**) Therefore, both scenarios (downregulation or upregulation of CMA) can result in malignancy. Figure created with Biorender.

Here, some solid examples have been presented that suggest that CMA can be implicated in tumor progression by controlling the cell cycle (Figure 3). CMA activity controls the protein levels of key molecules involved in the cell cycle, during processes in which cell proliferation must be modulated, such as malignant transformation, hypoxia, and response to DNA damage. In physiological conditions (Figure 3A), the activity of CMA governs the balance between positive and negative cell cycle regulators, providing an efficient cellular control mechanism of cell proliferation rates. However, whether CMA activity is deregulated, either downregulated (Figure 3B) or upregulated (Figure 3C), cellular malignancy is potentially induced. However, the actual effect of CMA in the cell cycle is still not clear, since this type of autophagy is able to regulate both positive and negative modulators of the cell cycle. Therefore, further studies are needed to understand the precise role of CMA in the cell cycle. Once CMA function in the control of the cell cycle, and its consequences in tumor development have been clarified, clinical implications arise. The results of these studies may be added to the evidence accumulated in the last decade that support the role of CMA in tumorigenesis. Thus, future research can reveal potential new therapeutic cancer strategies targeting CMA.
